# 2D SIFt: a matrix of ligand-receptor interactions

**DOI:** 10.1186/s13321-021-00545-9

**Published:** 2021-09-08

**Authors:** Stefan Mordalski, Agnieszka Wojtuch, Igor Podolak, Rafał Kurczab, Andrzej J. Bojarski

**Affiliations:** 1 Department of Medicinal Chemistry, Maj Institute of Pharmacology Polish Academy of Sciences, Krakow, Poland; 2 Faculty of Mathematics and Computer Science, Jagiellonian University, Krakow, Poland

**Keywords:** Structural Interaction Fingerprints, Ligand-receptor interactions, Fingerprints

## Abstract

Depicting a ligand-receptor complex via Interaction Fingerprints has been shown to be both a viable data visualization and an analysis tool. The spectrum of its applications ranges from simple visualization of the binding site through analysis of molecular dynamics runs, to the evaluation of the homology models and virtual screening. Here we present a novel tool derived from the Structural Interaction Fingerprints providing a detailed and unique insight into the interactions between receptor and specific regions of the ligand (grouped into pharmacophore features) in the form of a matrix, a 2D-SIFt descriptor. The provided implementation is easy to use and extends the python library, allowing the generation of interaction matrices and their manipulation (reading and writing as well as producing the average 2D-SIFt). The library for handling the interaction matrices is available via repository http://bitbucket.org/zchl/sift2d.

**Supplementary Information:**

The online version contains supplementary material available at 10.1186/s13321-021-00545-9.

## Introduction

Structural Interaction Fingerprints (SIFts), as described by Deng et al.[1], comprise a method for encrypting protein–ligand interactions in the form of a bit string. Such a fingerprint contains repeatable portions of a fixed length, with each position encoding a defined type of interaction. Our previous implementation of SIFts [2] employed nine types of interactions: any, with backbone, with side chain, hydrophobic, charged, hydrogen bond donor and acceptor, polar and aromatic, repeated for every residue in the protein. Interaction fingerprints are protein-centric, i.e., all parts of the fingerprint are encoded with respect to the residues alone. The ligand is, in fact, treated as a homogenous object to some extent because it is not possible to retrieve any information regarding its structure from the fingerprint.

SIFt belongs to the family of interaction fingerprints (IFP) [3] comprising a number of approaches to depict the protein–ligand interactions, ranging from ligand- to target-centric approaches. Ligand-based methods encode the interaction data into the ligand descriptor, forming a three-dimensional model resembling a pharmacophore [4], reducing the ligand structure to a set of interacting fragments [5–7] or enriching the compound structure with interaction data [8]. Target-oriented descriptors mostly follow the initial Deng concept [1], expanding the spectrum of the detected interactions [9, 10] and providing with novel visualization methods allowing easy visual inspection [11].

Finally, there is a number of IFP utilizing hybrid approach to the description of the protein–ligand complex, e.g. in a form of atom pairs [12, 13] or Extended Connectivity Interaction Features [14].

The utility of IFP as well as availability of numerous crystal structures enabled the assembly of databases containing structures annotated with interaction fingerprints [15–18], providing means to visualize binding pockets or screen using pre-generated interaction fingerprints Combining IFPs with generative machine learning models allows for prediction of the interaction fingerprint and docking score without the need of computationally expensive calculations[19].

Interaction-based approaches have been shown to be very potent tools for post-docking analysis of Virtual Screening (VS) results [9, 10, 20–23], performing comparably or better than classical ligand-based methods, for scoring the ligand-receptor complexes [24]. For instance, IFP has shown its potential to analyze the activity cliffs [22, 25], allowing identification of novel, potent ligands. In addition, the form of the binding site description with interaction fingerprints enables its easy visualization. Moreover, the use of descriptors that are independent of the ligand structure allows the identification of structurally new compounds, which is of extreme importance for VS campaigns aimed at the discovery of new potential drugs.

Bearing in mind the advantages of the interaction-based description of a ligand-receptor complex, we wanted to enrich the algorithm of SIFt generation with more detailed information about the ligand while maintaining the notation independent of the explicit ligand structure. This process resulted in the development of the 2D-SIFt descriptor (an interaction matrix), a matrix that depicts interactions per residue, not solely with the ligand treated as an object with uniform properties but with its different pharmacophore features. Analogously to the linear SIFt descriptor, the interaction matrix results from the concatenation of submatrices of a fixed size that encode interactions with individual amino acids. The developed descriptor maintains the potential for the rapid portrayal of the binding site, allowing easy extraction of the interacting residues; however, the use of ligand pharmacophore features allows the construction of heat maps of binding sites, which facilitate the identification of interaction hotspots (residues and dominating interaction types). A collection of individual interaction matrices can be averaged into a profile, providing a generalized overview of the binding mode and, concomitantly, of the common pharmacophore features of the interacting ligands.

This paper presents the python library for generating and manipulation of the 2D-SIFt descriptor along with two case studies, demonstrating key advantages of extending classical interaction fingerprints: rapid binding site description by means of an averaged descriptor, and the identification of key residues for binding of different types of ligands. In addition, we show that the modularity and flexibility of the interaction matrix format is easy to use with generic residue numbers (here: generic residue numbers for G protein-coupled receptors [26]). The targets of choice for the study are the G protein-coupled receptors (GPCRs) [27], being a superfamily of membrane proteins of great pharmacological relevance [28]. GPCRs share a common heptahelical topology of the transmembrane domain, encouraging the application of generic numbering schemes that describe the positions of the residues within the 7TM bundle rather than subsequent sequence numbers and thus permitting comparison between different members of the protein family [26].

## Implementation

**Fig. 1:**
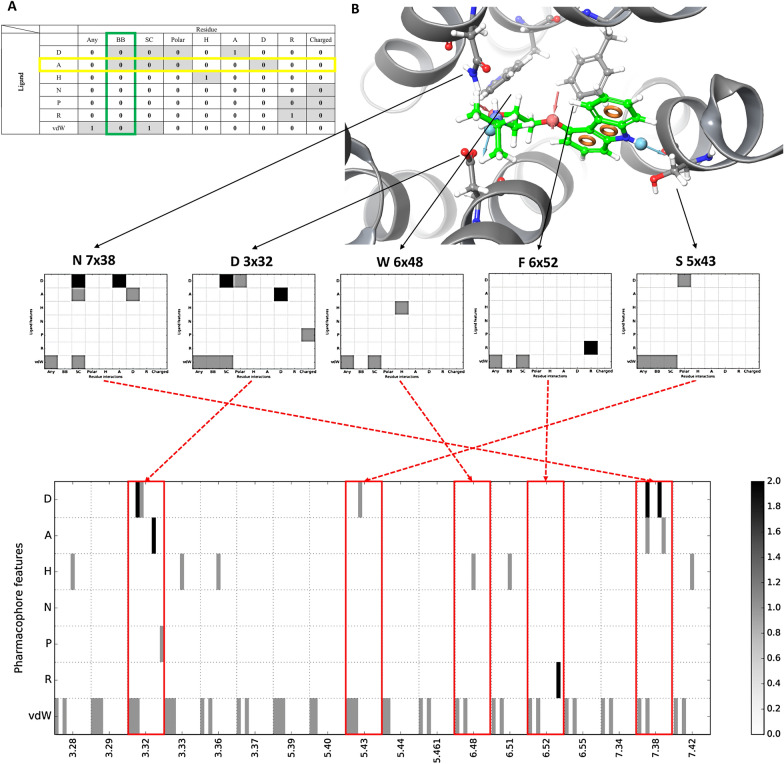
Scheme of construction of the 2D-SIFt descriptor. **A** Schematic representation of the 2D-SIFt portion depicting the interactions of one amino acid. Grayed fields show the incrementable bits. **B** The symbols in the column headers of the table describe the types of interactions: Any, *BB *with a backbone, *SC *interaction with a sidechain, *P* polar, *H* hydrophobic, *A* hydrogen bond acceptor, *D* hydrogen bond donor, *C* charged interaction, *R* aromatic. Rows denote standard pharmacophore features of the ligand: *A* hydrogen bond acceptor, *D* hydrogen bond donor, *H* hydrophobic, *N* negatively charged group, *P* positively charged group, *R* aromatic, *vdW* any atom. Each ligand-residue interaction is encoded as a 7 × 9 matrix of contacts, and the intensity of the gray corresponds to the number of features interacting with a ligand. Subsequent per-residue matrices are concatenated to form the 2D-SIFt descriptor. The structure presented is the crystal structure of a β2-adrenoreceptor receptor solved with inverse agonist, carazolol (2RH1)

The 2D-SIFt descriptor is a matrix of 7 × (9∙N) fields (six standard pharmacophore features together with a “wildcard” feature, nine types of interactions with amino acids, N —number of residues, Fig. 1A). The matrix fields can have values greater than 1 because there can be more than one separate pharmacophore feature of a given type within the ligand interacting with one residue (e.g. a number of aromatic rings surrounding a phenylalanine, Fig. 1B). The pharmacophore features used for the construction of the interaction matrix are common to every pharmacophore modeling software: hydrogen bond donor, hydrogen bond acceptor, hydrophobic group, negatively charged group, positively charged group, aromatic ring. In this research, the features were assigned by fitting the SMARTS patterns, as defined in RDKit library (http://www.rdkit.org, https://github.com/rdkit/rdkit/blob/master/Data/BaseFeatures.fdef). The collection of residual contacts consists of the previously described types: any, side chain, backbone, hydrogen bond donor, hydrogen bond acceptor, charged, hydrophobic and aromatic [2].

In the first step of the algorithm, the pharmacophore features are assigned to the ligand using the appropriate SMARTS patterns. Then, for every amino acid in the analyzed complex, the interactions between those features and the given residue are evaluated and encrypted into the fields of the interaction matrix. The concatenation of per-residue matrices results in a 2D-SIFt interaction matrix (Fig. 1B).

The interactions are evaluated in a similar manner to one-dimensional SIFt [2]: residues are grouped into classes: aromatic, hydrophobic, polar, negatively and positively charged. For charged, hydrophobic and vdW contacts, the distance (here no greater than 3.5 Å) and the complementarity of interacting features is the basis of detection of the interaction. However, for hydrogen bonds, the maximum distance is 2.8 Å and in addition, the donor (Y-H…X, where X and Y are heavy atoms, H—hydrogen) must not exceed 120° and acceptor (X…H-Y) 90°. For π interactions, the distances are 4.4 Å, 5.5 Å and 6.6 Å for face to face, edge to face and π-cation interactions, respectively. Maximum angle between the aromatic rings in face to face interactions was of 30° and the minimum angle in edge to face was 60°. To avoid redundancy of information, a set of rules was introduced to guide the incrementation of bits in the interaction matrix for one residue.

The interaction profiles are calculated by averaging every field of the interaction matrix. However, to avoid noise and obtain a clear dominating binding mode, values lower than the established threshold (0.3 for the case studies described herein) are silenced (set to 0).

The algorithm was implemented in Python using the RDKit library (http://www.rdkit.org). Curated (cleaned and annotated with generic numbers) crystal structures of GPCRs were obtained from GPCRdb [29]. Protein Preparation Wizard was used to prepare the receptor structures (assignment of the bond order, atom types and optimization of the hydrogen bond networks).

### Case study: common antagonist binding site for Class A GPCRs

As previously indicated, the generic numbering scheme for the transmembrane residues of the GPCRs allows comparison between different proteins within the family. This approach has been utilized previously [30] to estimate common binding site for the Class A GPCRs based on crystallographic data. In this case study, analysis of the binding modes was conducted based solely on the crystalline ligand-receptor complexes obtained from GPCRdb [29]. A set of 33 unique Class A targets crystalized with small molecules in the presumably inactive conformation (no G protein nor nanobody bound) was taken into the study. For multiple crystals with different ligands, the antagonists were of preference, however, in case of serotonin receptors 1B and 2B, only agonist-bound structures were available. Among crystals of the same target with antagonists, the structure of the best resolution was used. For a comparison, a similar study was performed on the set of 154 crystal structures of the Class A GPCRs co-crystalized small molecule ligand (out of 173 Class A crystals annotated in GPCRdb in June 2017).

### Case study: agonist and antagonist binding modes for β_2_-AR

The comparison of the binding modes for antagonists and agonists was based on the interaction matrices (2D-SIFt) generated for nine complexes of β_2_**-**AR (Additional file 1: Table S2) [31–36]. To provide the consistency of the structural data, agonists binding mode was analyzed for the active structures of the β_2_**-**AR. The conformation was assumed active whenever the receptor was bound to an agonist along with a cofactor stabilizing the active state, either a G protein (3SN6) or a nanobody. In addition, the crystal structure of β_2_**-**AR with irreversible agonist was included into the study. In the case of multiple crystals with an identical ligand, a structure with higher resolution was selected for the study. An inactive receptor was defined as a structure with either an antagonist or an inverse agonist bound. Analogously, a higher resolution was preferred for multiple structures that crystalized with the same compound.

## Results

### Case study: common antagonist binding site for G protein-coupled receptors

Analysis of the binding sites resulted in 67 GPCRdb residue positions [26] interacting with ligands; however, a low interaction frequency was observed for most of them. Setting the contact threshold to more than 30% of the crystals resulted in 21 positions (Table 1, Additional file 1: Fig. S1) that were hotspots for ligand-receptor interactions. The residue positions forming the most frequent contacts, 3 × 32, 3 × 33, 3 × 36, 6 × 48, 6 × 51 and 7 × 38 (corresponding to 7.39 in Ballesteros-Weinstein notation [37]), occurring in over 70% structures, form previously reported [30] consensus framework for interactions with ligands across Class A GPCRs. Here, additional positions, 5 × 43, 5 × 461, 6 × 55 and 7 × 42, also appeared as ligand anchors (scoring at least 70% contact frequency). All those residue positions were also identified as Orthosteric Binding Site (OBS) [38]. Interestingly, the study also revealed the populations of residue positions belonging to the Secondary Binding Pockets (SBPs) [38], spanning TM2 (2 × 60, 2 × 63) and TM7 (7 × 34, 7 × 35 and 7 × 41) and being the extension of the OBS. Secondary binding pockets play a role in subtype selectivity, as the SBP residues are less conserved. Indeed, 2D-SIFt descriptors indicated distinct interaction profiles within secondary pockets for different receptor subtypes (e.g. opioid receptors, angiotensin receptor or muscarinic receptors—Table 1). Tabular visualization of the interacting ligand features also facilitated the selection of outliers—receptors accommodating distinct type of ligands and showing distinct interaction pattern (LPA_1_ receptor, 4Z35) or having unusually located binding site (e.g. P2Y_1_ receptor, 4XNV, binding ligand outside of the OBS).

**Table 1:**
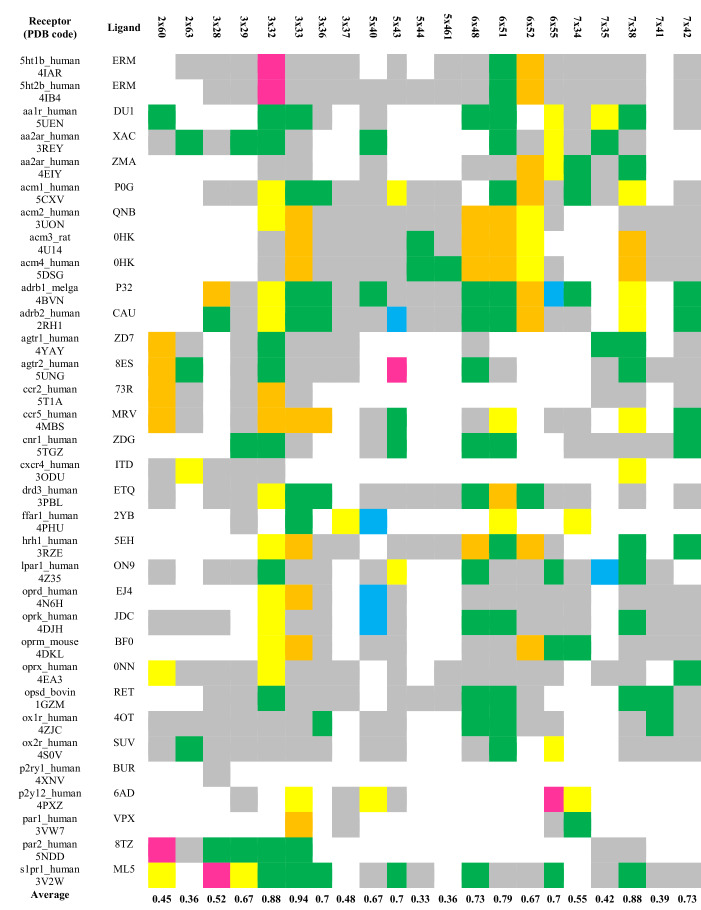
The most frequent interactions within the common binding site of the GPCRs crystallized with small molecule ligands (representative structures). Colors correspond to the specific interaction formed by the ligand pharmacophore features: purple, charged; yellow, hydrogen bond acceptor; blue, hydrogen bond donor; green, hydrophobic; orange, aromatic; gray, any

Similar analysis performed for the full set of available crystal structures of GPCRs bound to small molecules resulted in 19 common interaction hotspots (Additional file 1: Table S1), lacking the TM2 residues identified for representative structures. Again, the set of the residue positions the most frequently interacting with ligands matches the one elucidated for representative structures (3 × 32, 3 × 33, 3 × 36, 5 × 43, 5 × 461, 6 × 48, 6 × 51, 6 × 55, 7 × 38 and 7 × 42 found to be interacting in over 70% crystals). On the other hand, the redundancy of the interactions in the full set of crystal structures (e.g. 17 crystals of β_2_-AR) does not allow detecting all of the secondary binding pockets but only provides the dominating interacting positions.

List of the common interacting residues was extracted from 35 distinct small molecule (antagonist)-bound crystal structures of GPCRs. Positions that interacted with less than 30% of the structures were excluded. Residue positions were encoded using a GPCRdb numbering scheme, and the contact frequency shows the fraction of detected interactions across the investigated dataset. Colors correspond to the specific interaction formed by the ligand pharmacophore features: purple, charged; yellow, hydrogen bond acceptor; blue, hydrogen bond donor; green, hydrophobic; orange, aromatic; gray, any.

### Case study: agonist and antagonist binding modes for β_2_-AR

**Fig. 2:**
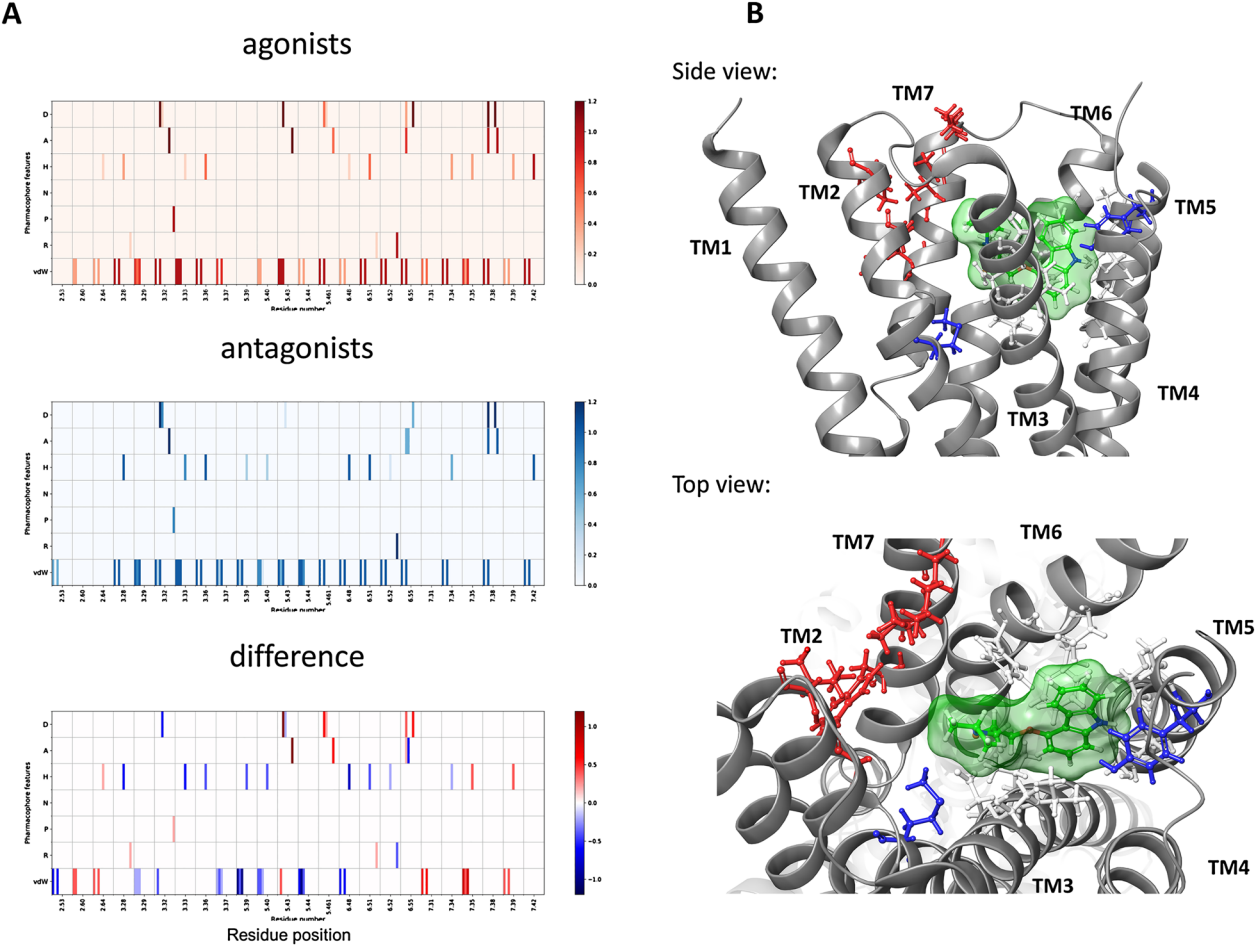
Differences between agonistic and antagonistic binding site for β_2_-AR. Depicted by the 2D-SIFt profile for agonists (red) and antagonists (blue) (**A**), and visualized in the crystal structure of β_2_-AR, 2RH1 (**B**). Residues in white are common to both binding modes, blue is unique to antagonist binding and red is unique to agonist binding. For clarity, the 2D-SIFt heatmaps display the per-residue interactions found in more than 30% of the investigated complexes

The 2D-SIFt descriptors, which were constructed for the dataset of five active and five inactive complexes (Additional file 1: Table S2), revealed differences in the binding modes of the ligand types in terms of both the composition of the set of interacting amino acids and the pharmacophore features of the interacting ligands (Fig. 2A). The common set of 24 residues encompasses the antagonist binding site, with two residues that are unique for the antagonist binding site (M82^2.53x53^ and Y199^5.38x39^) and five for the agonist binding site (G90^2.61x60^, I94^2.65x64^, K305^7.30x31^, I309^7.36x35^, W313^7.40x39^_,_ Fig. 2B). However, the differences consist not only of the spatial placement of the binding sites—the set of common interacting residues, but also the prevalent types of interactions that are distinct for different types of ligand. Antagonists tend to form less specific interactions, with van der Waals and hydrophobic contacts as the dominating mode of interaction. Agonists, in contrast, often interact via hydrogen bonds and polar contacts, as shown in the differential profile (D113^3.32x32^, S204^5.42x43^, S207^5.46x461^ and N312^7.39x38^, Fig. 2A). These differences can be explained by the pharmacological role of the compounds: agonists induce a significant change in receptor conformation. This observation for the amino acid composition and specific interactions may serve as a simple filter to distinguish the role of the ligand based solely on the interaction matrix.

The analysis performed for the extended set of all available agonist and antagonist bound structures resulted in narrower set of common residue positions, omitting the TM2 contacts (Additional file 2: Fig. S2). The differences in specific interactions for the remaining positions, however, remained nearly identical.

### 1D representation

Although matrix representation of the 2D-SIFt descriptor improves visual inspection and analysis of the ligand-receptor interactions, converting the descriptor into bit string would allow for use with existing tools for fingerprint analysis. This would facilitate docking postprocessing or filtering the docking poses with minimal effort. For this reason, the implementation of the 2D-SIFt comes with functions to convert a 2D-SIFt matrix onto linear representation either allowing for values greater than 1 for interactions or keeping the descriptor binary. We have also provided simple tools for calculating both, Tanimoto and Euclidean distance between fingerprints, for binary and non-binary representation, respectively. Linear descriptor contains all incrementable bits from 2D-SIFt matrix (Fig. 1A).

## Conclusions

The library presented herein for the automated generation and manipulation of the 2D-SIFt descriptor along with convenient visualization in the form of a heat map provides a powerful tool to consolidate the interaction data obtained from multiple ligand-receptor complexes. As shown in the case studies, the toolkit supports a generic numbering scheme for proteins, thus allowing the assessment of structures with differing sequences but similarities within the same numbering space. The intuitive interpretation of the visualized descriptors (heat maps) provides the opportunity to identify interaction hotspots easily, as well as to differentiate between the binding modes associated with compounds with different pharmacological roles. The visualization in form of a table allows for quick assessment of contributions of individual complexes to the interaction profile, and thus quick identification of the ligand-receptor complexes not matching the common interaction pattern. The 2D-SIFt interaction matrix, which combines both structural and pharmacophore data, supplies a hybrid structure- and ligand-based analysis. The automated approach to the interaction analysis provides convergent results with the implementation of a meticulous manual analysis, yet it is convenient to use and customizable.

## Supplementary Information

**Additional file 1**: **Figure S1.** A 2D-SIFt representation of the common binding site profile for the antagonists in G Protein-Coupled Receptors. The intensity of gray corresponds to the number of feature-receptor interactions. **Table S1.** Crystal structures used for the construction of the common antagonist binding site for Class A GPCRs. **Table S2.** Crystal structures used for the comparison of the agonist and antagonist binding modes for β2AR. **Table S3.** Individual 2D-SIFt heat maps for the crystal structures used for construction of the common binding site of the GPCR antagonists.

**Additional file 2**: **Figure S2**.

## Data Availability

Project name: 2D-SIFt. Project home page: https://bitbucket.org/zchl/sift2d Operating system(s): Platform independent. Programming language: Python 3. Other requirements: Commercial packages supplied by Schrodinger LLC (free for academic users). License: GPL. Any restrictions to use by non-academics: Third party library.

## References

[1] Deng Z, Chuaqui C, Singh J (2004). Structural interaction fingerprint (SIFt): a novel method for analyzing three-dimensional protein-ligand binding interactions. J Med Chem.

[2] Mordalski S, Kosciolek T, Kristiansen K, Sylte I, Bojarski AJAJ (2011). Protein binding site analysis by means of structural interaction fingerprint patterns. Bioorg Med Chem Lett.

[3] Salentin S, Haupt VJ, Daminelli S, Schroeder M (2014). Polypharmacology rescored: protein-ligand interaction profiles for remote binding site similarity assessment. Prog Biophys Mol Biol.

[4] Wolber G, Langer T (2005). LigandScout: 3-D pharmacophores derived from protein-bound ligands and their use as virtual screening filters. J Chem Inf Model.

[5] Tan L, Lounkine E, Bajorath J (2008). Similarity searching using fingerprints of molecular fragments involved in protein-ligand interactions. J Chem Inf Model.

[6] Tan L, Vogt M, Bajorath J (2009). Three-dimensional protein-ligand interaction scaling of two-dimensional fingerprints. Chem Biol Drug Des.

[7] Tan L, Bajorath J (2009). Utilizing target-ligand interaction information in fingerprint searching for ligands of related targets. Chem Biol Drug Des.

[8] Crisman TJ, Sisay MT, Bajorath J (2008). Ligand-target interaction-based weighting of substructures for virtual screening. J Chem Inf Model.

[9] Marcou G, Rognan D (2007). Optimizing fragment and scaffold docking by use of molecular interaction fingerprints. J Chem Inf Model.

[10] Sato T, Honma T, Yokoyama S (2010). Combining machine learning and pharmacophore-based interaction fingerprint for in silico screening. J Chem Inf Model.

[11] Clark AM, Labute P (2007). 2D depiction of protein-ligand complexes. J Chem Inf Model.

[12] Desaphy J, Raimbaud E, Ducrot P, Rognan D (2013). Encoding protein-ligand interaction patterns in fingerprints and graphs. J Chem Inf Model.

[13] Pérez-Nueno VI, Rabal O, Borrell JI, Teixidó J (2009). APIF: a new interaction fingerprint based on atom pairs and its application to virtual screening. J Chem Inf Model.

[14] Sánchez-Cruz N, Medina-Franco JL, Mestres J, Barril X (2021). Extended connectivity interaction features: improving binding affinity prediction through chemical description. Bioinformatics.

[15] Kooistra AJ, Kanev GK, van Linden OPJ, Leurs R, de Esch IJP, de Graaf C (2016). KLIFS: a structural kinase-ligand interaction database. Nucleic Acids Res.

[16] Desaphy J, Bret G, Rognan D, Kellenberger E (2015). sc-PDB: a 3D-database of ligandable binding sites–10 years on. Nucleic Acids Res.

[17] Kufareva I, Ilatovskiy AV, Abagyan R (2012). Pocketome: an encyclopedia of small-molecule binding sites in 4D. Nucleic Acids Res.

[18] Schreyer AM, Blundell TL (2013). CREDO: a structural interactomics database for drug discovery. Database (Oxford)..

[19] Jastrzębski S, Szymczak M, Pocha A, Mordalski S, Tabor J, Bojarski AJ (2020). Emulating docking results using a deep neural network: a new perspective for virtual screening. J Chem Inf Model.

[20] Mordalski S, Witek J, Smusz S, Rataj K, Bojarski AJ (2015). Multiple conformational states in retrospective virtual screening—homology models vs. crystal structures: beta-2 adrenergic receptor case study. J Cheminform..

[21] Witek J, Smusz S, Rataj K, Mordalski S, Bojarski AJ (2013). An application of machine learning methods to structural interaction fingerprints-a case study of kinase inhibitors. Bioorg Med Chem Lett.

[22] Méndez-Lucio O, Kooistra AJ, De GC, Bender A, Medina-Franco JL (2015). Analyzing multitarget activity landscapes using protein-Ligand interaction fingerprints: interaction cliffs. J Chem Inf Model.

[23] de Graaf C, Kooistra AJ, Vischer HF, Katritch V, Kuijer M, Shiroishi M (2011). Crystal structure-based virtual screening for fragment-like ligands of the human histamine H(1) receptor. J Med Chem.

[24] Yang L, Yang G, Chen X, Yang Q, Yao X, Bing Z (2021). Deep scoring neural network replacing the scoring function components to improve the performance of structure-based molecular docking. ACS Chem Neurosci.

[25] Stumpfe D, Hu Y, Dimova D, Bajorath J (2014). Recent progress in understanding activity cliffs and their utility in medicinal chemistry. J Med Chem.

[26] Isberg V, De Graaf C, Bortolato A, Cherezov V, Katritch V, Marshall FHFH (2015). Generic GPCR residue numbers—aligning topology maps while minding the gaps. Trends Pharmacol Sci.

[27] Overington JP, Al-Lazikani B, Hopkins AL (2006). How many drug targets are there?. Nat Rev Drug Discov.

[28] Heng BC, Aubel D, Fussenegger M (2013). An overview of the diverse roles of G-protein coupled receptors (GPCRs) in the pathophysiology of various human diseases. Biotechnol Adv.

[29] Isberg V, Mordalski S, Munk C, Rataj K, Harpsøe K, Hauser AS (2015). GPCRdb: an information system for G protein-coupled receptors. Nucleic Acids Res.

[30] Venkatakrishnan AJ, Deupi X, Lebon G, Tate CG, Schertler GF, Babu MM (2013). Molecular signatures of G-protein-coupled receptors. Nature.

[31] Ring AM, Manglik A, Kruse AC, Enos MD, Weis WI, Garcia KC (2013). Adrenaline-activated structure of β2-adrenoceptor stabilized by an engineered nanobody. Nature.

[32] Weichert D, Kruse AC, Manglik A, Hiller C, Zhang C, Hübner H (2014). Covalent agonists for studying G protein-coupled receptor activation. Proc Natl Acad Sci U S A.

[33] Cherezov V, Rosenbaum DM, Hanson MA, Rasmussen SGF, Thian FS, Kobilka TS (2007). High-resolution crystal structure of an engineered human beta2-adrenergic G protein-coupled receptor. Science.

[34] Hanson MA, Cherezov V, Griffith MT, Roth CB, Jaakola V-P, Chien EYT (2008). A specific cholesterol binding site is established by the 2.8 A structure of the human beta2-adrenergic receptor. Structure.

[35] Wacker D, Fenalti G, Brown MA, Katritch V, Abagyan R, Cherezov V (2010). Conserved binding mode of human beta2 adrenergic receptor inverse agonists and antagonist revealed by X-ray crystallography. J Am Chem Soc.

[36] Rosenbaum DM, Zhang C, Lyons JA, Holl R, Aragao D, Arlow DH (2011). Structure and function of an irreversible agonist-β(2) adrenoceptor complex. Nature.

[37] Ballesteros JA, Weinstein H (1995). Receptor molecular biology. Methods in neurosciences.

[38] Michino M, Beuming T, Donthamsetti P, Newman AH, Javitch JA, Shi L (2014). What can crystal structures of aminergic receptors tell us about designing subtype-selective ligands?. Pharmacol Rev.

