# Supplementary material for: 2D SIFt: a matrix of ligand-receptor interactions
Source: J Cheminform. 2021 Sep 8;13:66. doi: 10.1186/s13321-021-00545-9 (PMC8424890; doi:10.1186/s13321-021-00545-9)
Supplement: Supplementary file 1 — Additional file 1: Figure S1. A 2D-SIFt representation of the common binding site profile for the antagonists in G Protein-Coupled Receptors. The intensity of gray corresponds to the number of feature-receptor interactions. Table S1. Crystal structures used for the construction of the common antagonist binding site for Class A GPCRs. Table S2. Crystal structures used for the comparison of the agonist and antagonist binding modes for β2AR. Table S3. Individual 2D-SIFt heat maps for the crystal structures used for construction of the common binding site of the GPCR antagonists. [file 13321_2021_545_MOESM1_ESM.doc]

**2D SIFt – a matrix of ligand-receptor interactions**

Stefan Mordalski^1*^, Agnieszka Pocha^2^, Igor Podolak^2^, Rafał Kurczab^1^, Andrzej J. Bojarski^1^

^1^Department of Medicinal Chemistry, Institute of Pharmacology Polish Academy of Sciences, Smetna 12, 31-343 Krakow, Poland

^2^Faculty of Mathematics and Computer Science, Jagiellonian University, Lojasiewicza 6, 30-348 Krakow, Poland


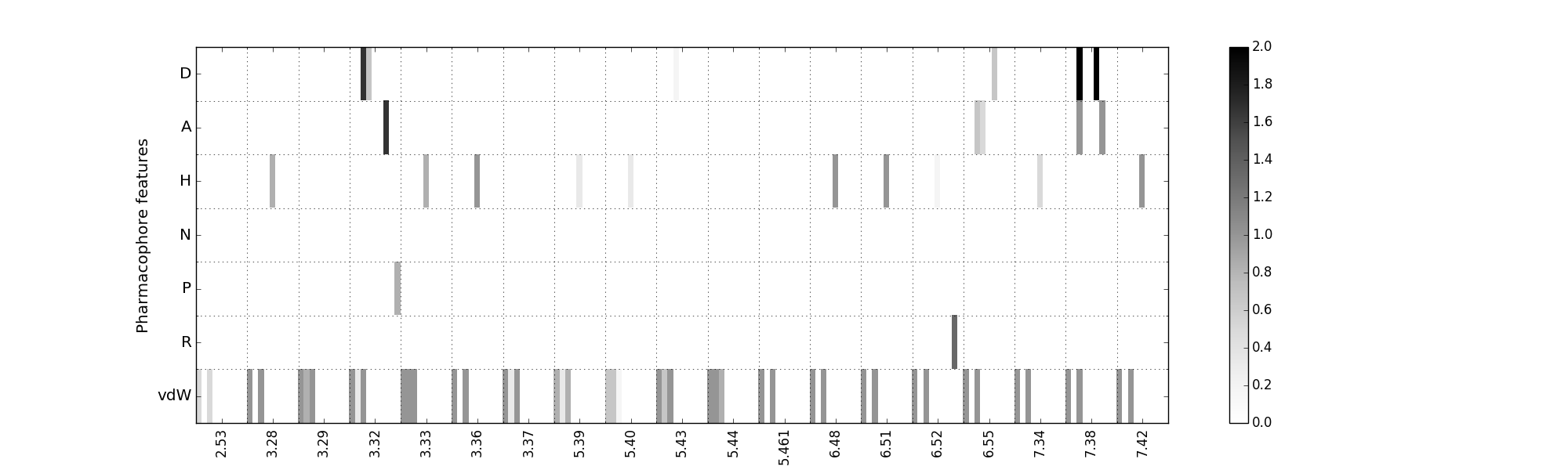


**Figure S1.** A 2D-SIFt representation of the common binding site profile for the antagonists in G Protein-Coupled Receptors. The intensity of gray corresponds to the number of feature-receptor interactions.

**Table S1**. Crystal structures used for the construction of the common antagonist binding site for Class A GPCRs.

| **PDB code** | **Structure title** | **Resolution [Å]** |
| --- | --- | --- |
| 1U19 | Crystal Structure of Bovine Rhodopsin at 2.2 Angstroms Resolution | 2.2 |
| 2RH1 | High resolution crystal structure of human B2-adrenergic G protein-coupled receptor. | 2.8 |
| 3ODU | The 2.5 A structure of the CXCR4 chemokine receptor in complex with small molecule antagonist IT1t | 2.5 |
| 3PBL | Structure of the human dopamine D3 receptor in complex with eticlopride | 2.9 |
| 3RZE | Structure of the human histamine H1 receptor in complex with doxepin | 3.1 |
| 3UON | Structure of the human M2 muscarinic acetylcholine receptor bound to an antagonist | 3.0 |
| 3V2Y | Crystal Structure of a Lipid G protein-Coupled Receptor at 2.80A | 2.8 |
| 3VW7 | Crystal structure of human protease-activated receptor 1 (PAR1) bound with antagonist vorapaxar at 2.2 angstrom | 2.2 |
| 4BVN | Ultra-thermostable beta1-adrenoceptor with cyanopindolol bound | 2.1 |
| 4DJH | Structure of the human kappa opioid receptor in complex with JDTic | 2.9 |
| 4DKL | Crystal structure of the mu-opioid receptor bound to a morphinan antagonist | 2.8 |
| 4EA3 | Structure of the N/OFQ Opioid Receptor in Complex with a Peptide Mimetic | 3.0 |
| 4EIY | Crystal structure of the chimeric protein of A2aAR-BRIL in complex with ZM241385 at 1.8A resolution | 1.8 |
| 4IAR | Crystal structure of the chimeric protein of 5-HT1B-BRIL in complex with ergotamine (PSI Community Target) | 2.7 |
| 4IB4 | Crystal structure of the chimeric protein of 5-HT2B-BRIL in complex with ergotamine | 2.7 |
| 4MBS | Crystal Structure of the CCR5 Chemokine Receptor | 2.7 |
| 4N6H | 1.8 A Structure of the human delta opioid 7TM receptor (PSI Community Target) | 1.8 |
| 4PHU | Crystal structure of Human GPR40 bound to allosteric agonist TAK-875 | 2.6 |
| 4PZX | Synthesis, Characterization and PK/PD Studies of a Series of Spirocyclic Pyranochromene BACE1 Inhibitors | 2.5 |
| 4S0V | Crystal structure of the human OX2 orexin receptor bound to the insomnia drug Suvorexant | 2.5 |
| 4U15 | M3-mT4L receptor bound to tiotropium | 2.8 |
| 4XNV | The human P2Y1 receptor in complex with BPTU | 2.2 |
| 4YAY | XFEL structure of human Angiotensin Receptor | 2.9 |
| 4Z35 | Crystal Structure of Human Lysophosphatidic Acid Receptor 1 in complex with ONO-9910539 | 2.9 |

**Table S2**. Crystal structures used for the comparison of the agonist and antagonist binding modes for β2AR.

|  | **PDB code** | **Structure title** | **Resolution [Å]** |
| --- | --- | --- | --- |
| **agonists** | 4LDE | Structure of beta2 adrenoceptor bound to BI167107 and an engineered nanobody | 2.8 |
|  | 4LDL | Structure of beta2 adrenoceptor bound to hydroxybenzylisoproterenol and an engineered nanobody | 3.1 |
|  | 4LDO | Structure of beta2 adrenoceptor bound to adrenaline and an engineered nanobody | 3.2 |
|  | 4QKX | Structure of beta2 adrenoceptor bound to a covalent agonist and an engineered nanobody | 3.3 |
| **antagonists** | 2RH1 | High resolution crystal structure of human B2-adrenergic G protein-coupled receptor. | 2.4 |
|  | 3D4S | Cholesterol bound form of human beta2 adrenergic receptor. | 2.8 |
|  | 3NY8 | Crystal structure of the human beta2 adrenergic receptor in complex with the inverse agonist ICI 118,551 | 2.8 |
|  | 3NY9 | Crystal structure of the human beta2 adrenergic receptor in complex with a novel inverse agonist | 2.8 |
|  | 3NYA | Crystal structure of the human beta2 adrenergic receptor in complex with the neutral antagonist alprenolol | 3.2 |

**Table S3.** Individual 2D-SIFt heat maps for the crystalstructures used for construction of the common binding site of the GPCR antagonists.

| Rhodopsin 1U19 | 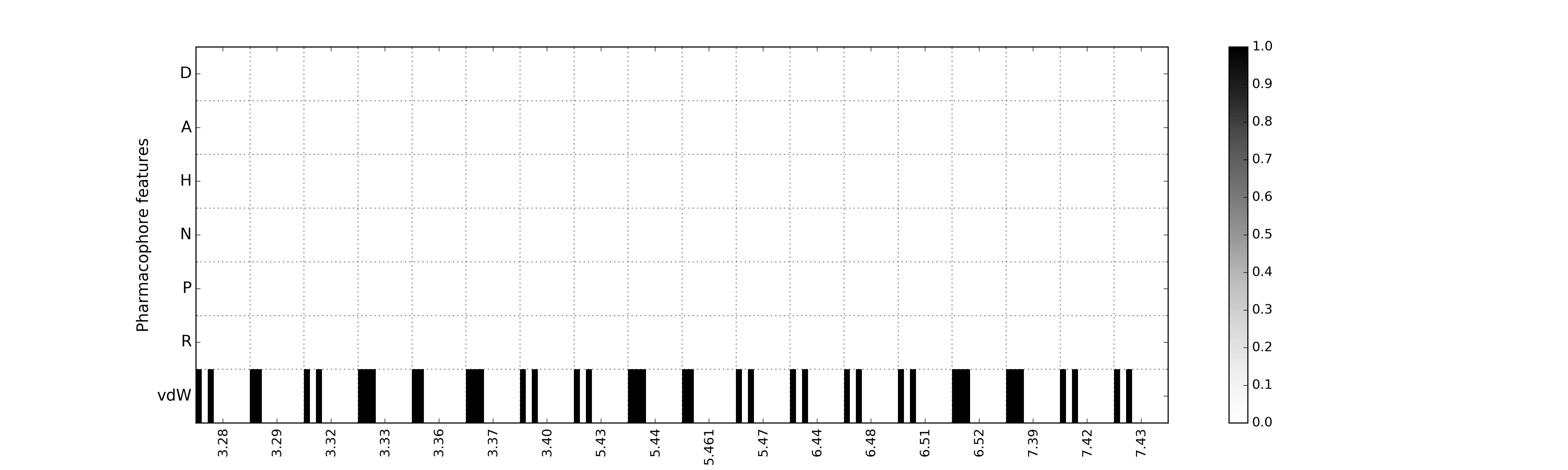 |
| --- | --- |
| β_2_-adrenoceptor 2RH1 | 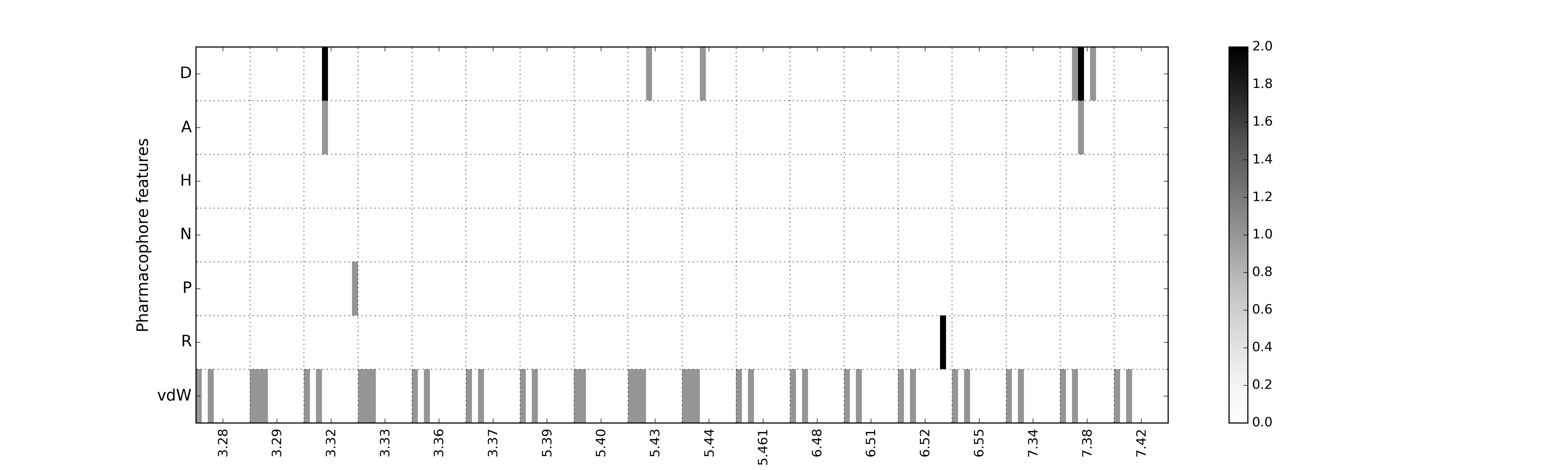 |
| CXCR4 3ODU | 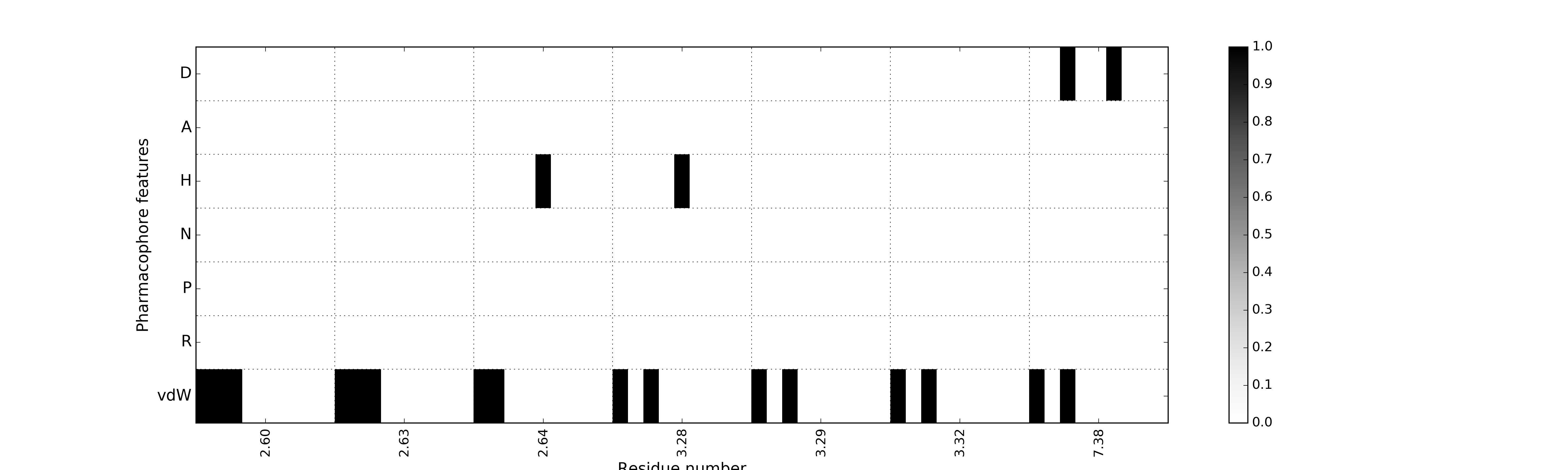 |
| D_3_ receptor 3PBL | 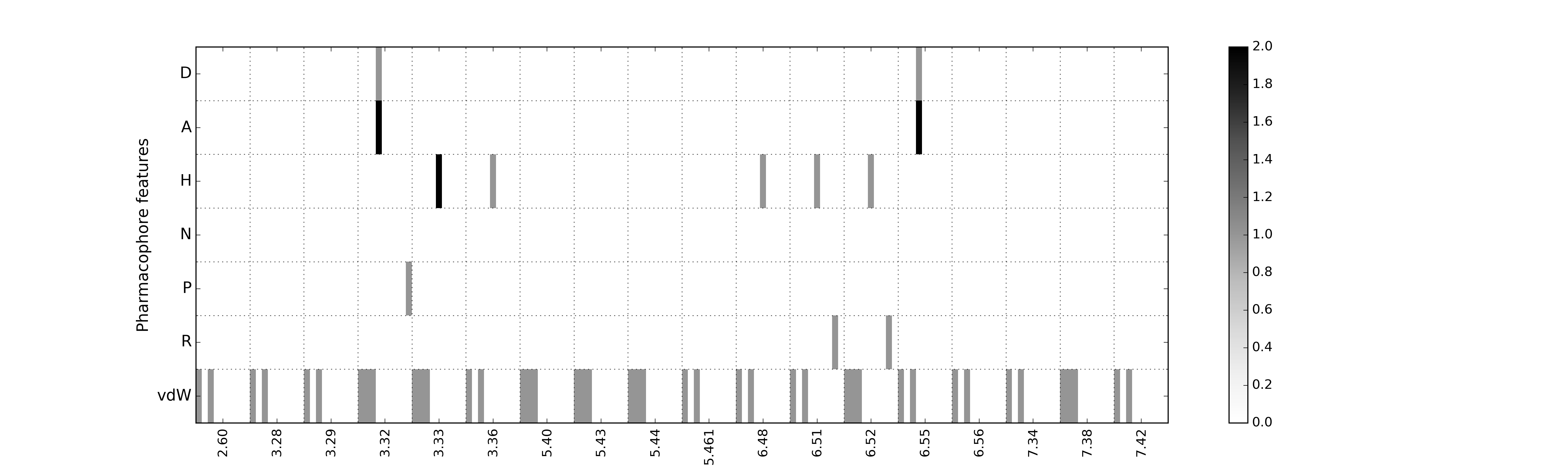 |
| H_1_ receptor 3RZE | 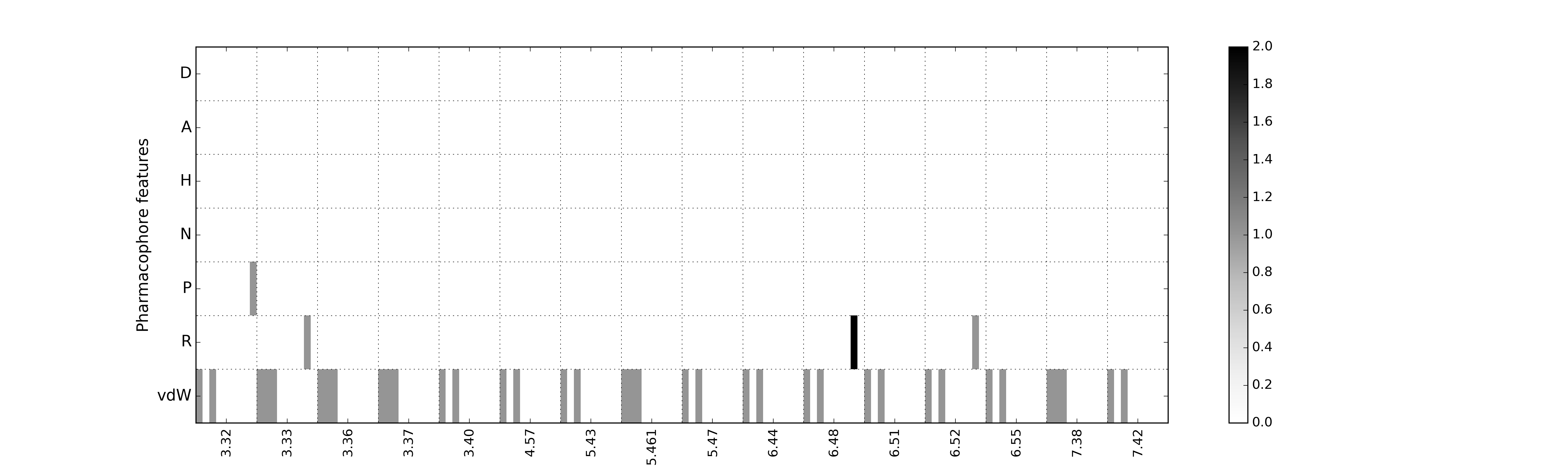 |
| M_2_ receptor 3UON | 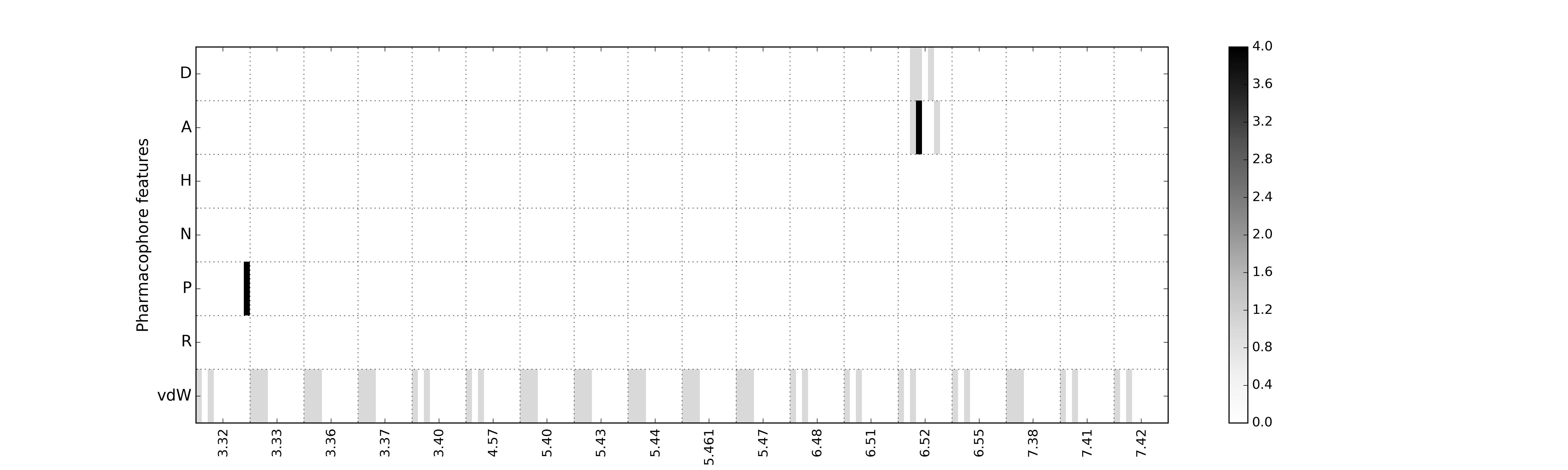 |
| S1P_1_ receptor 3V2Y | 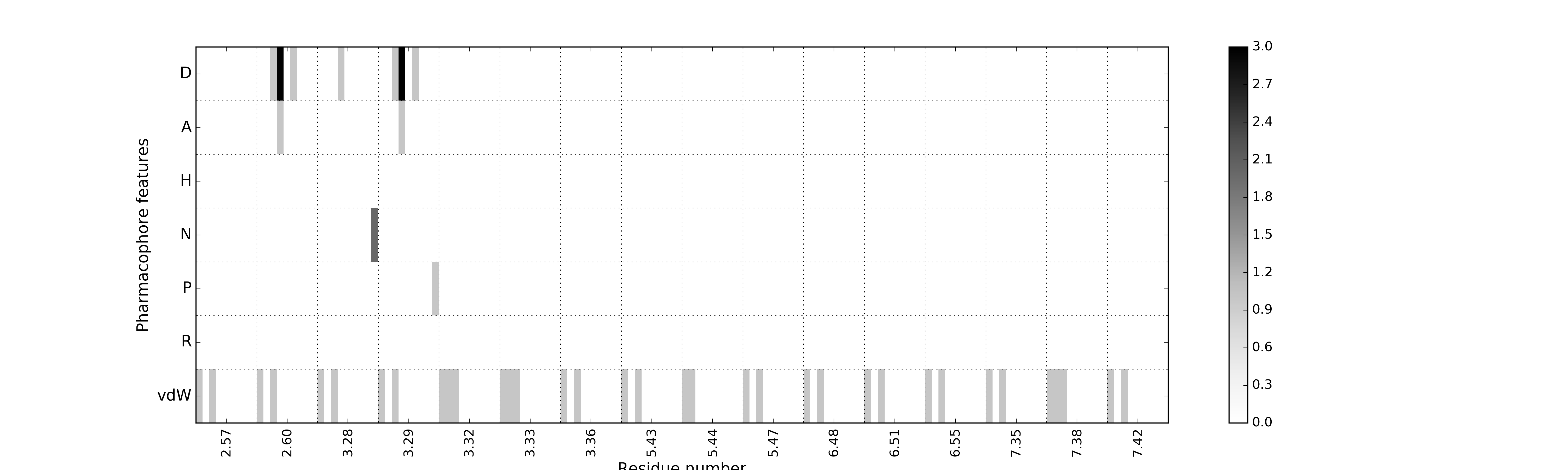 |
| PAR1 3VW7 | 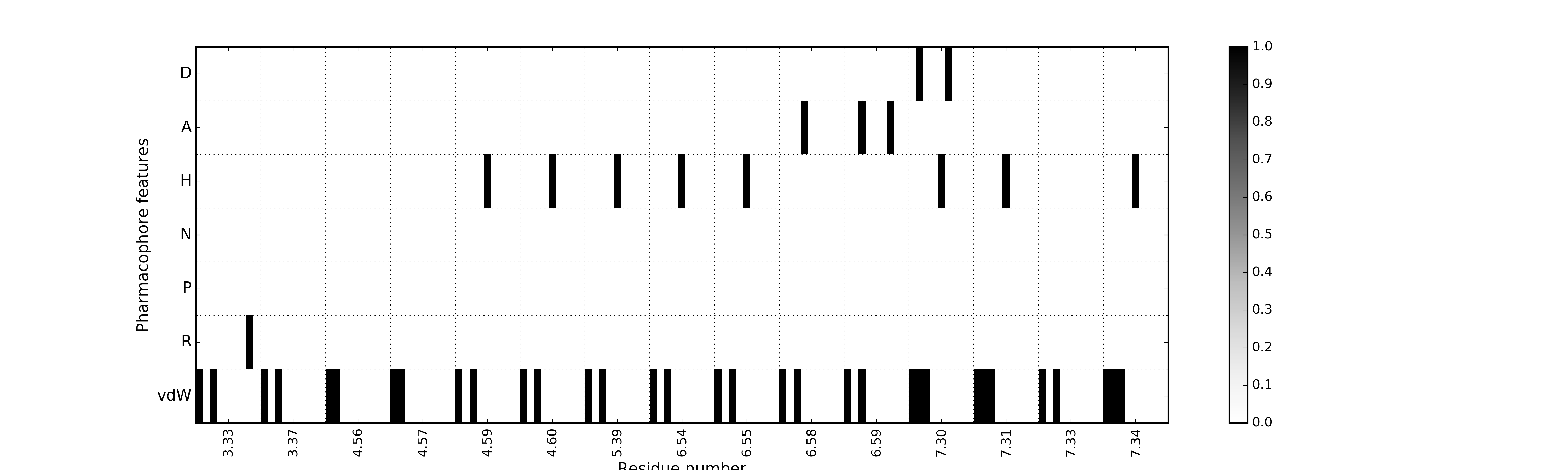 |
| β_1_-adrenoreceptor 4BVN | 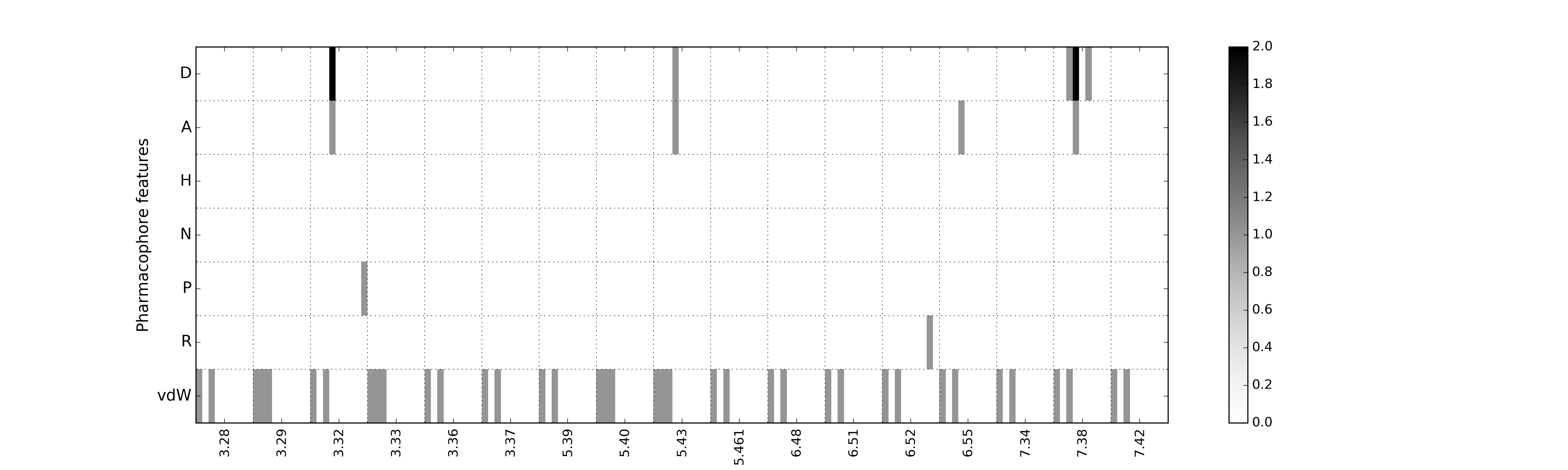 |
| κ receptor 4DJH | 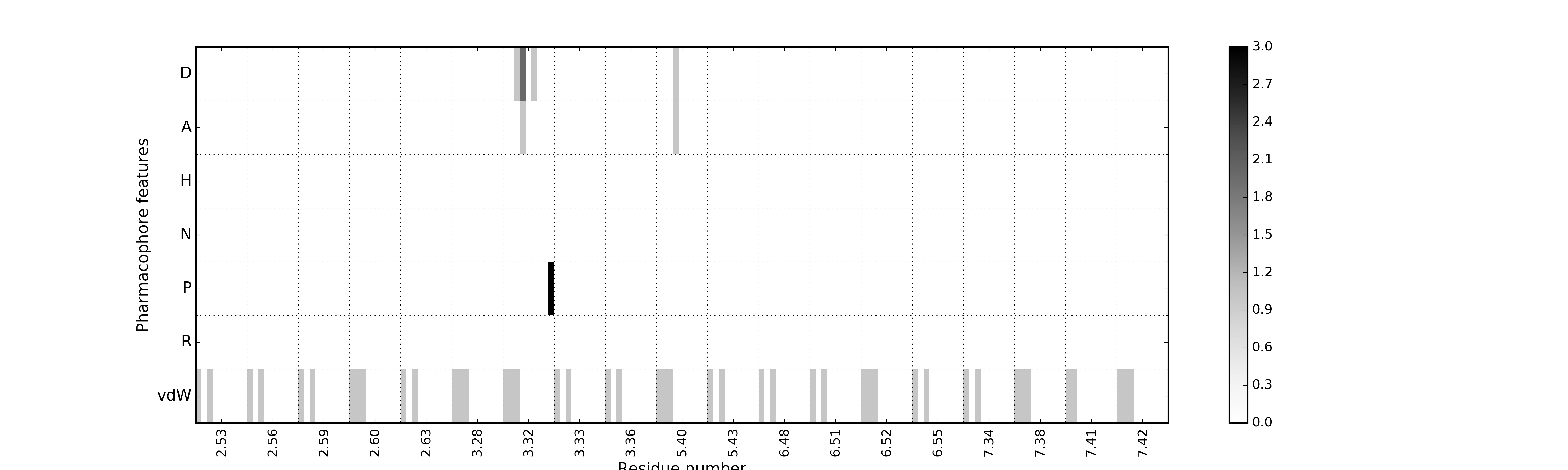 |
| μ receptor 4DKL | 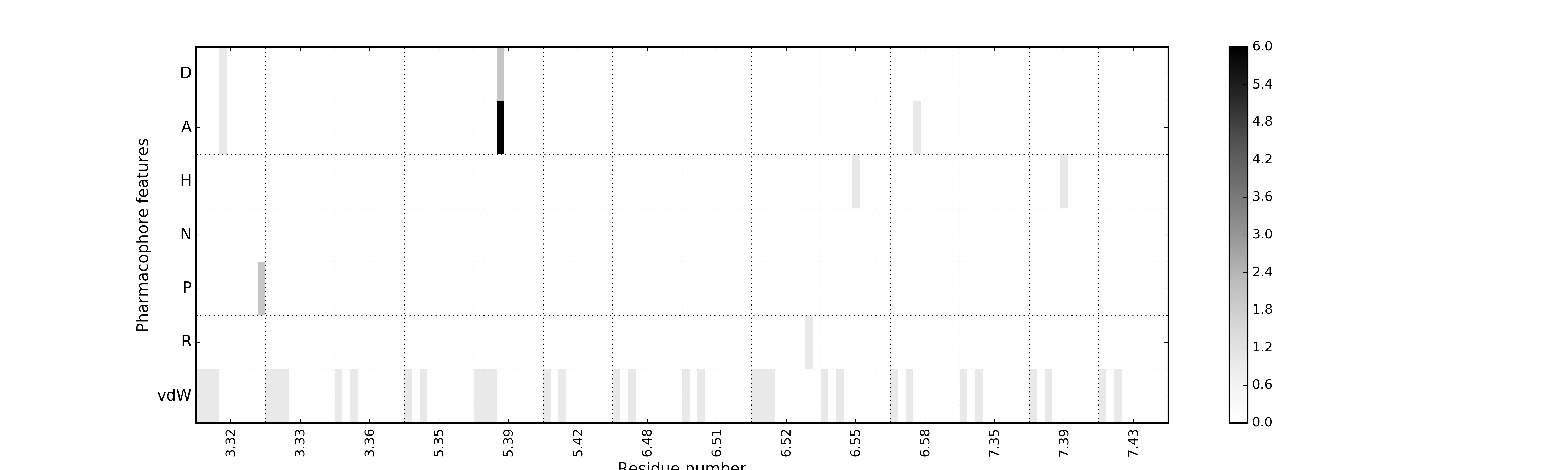 |
| NOP receptor 4EA3 | 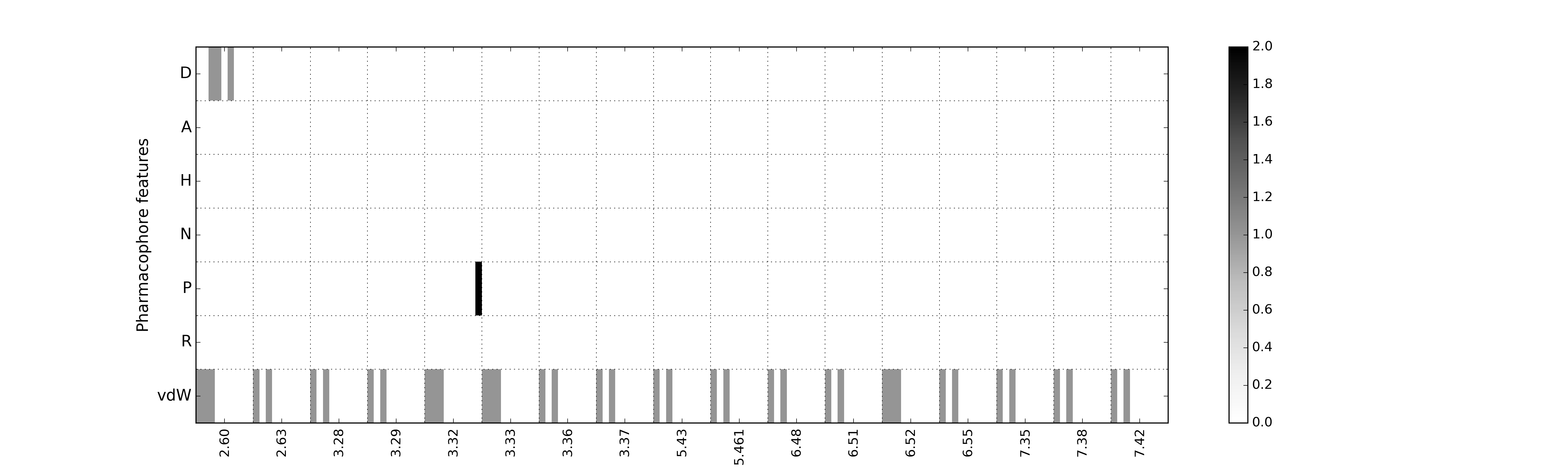 |
| A_2A_ receptor 4EIY | 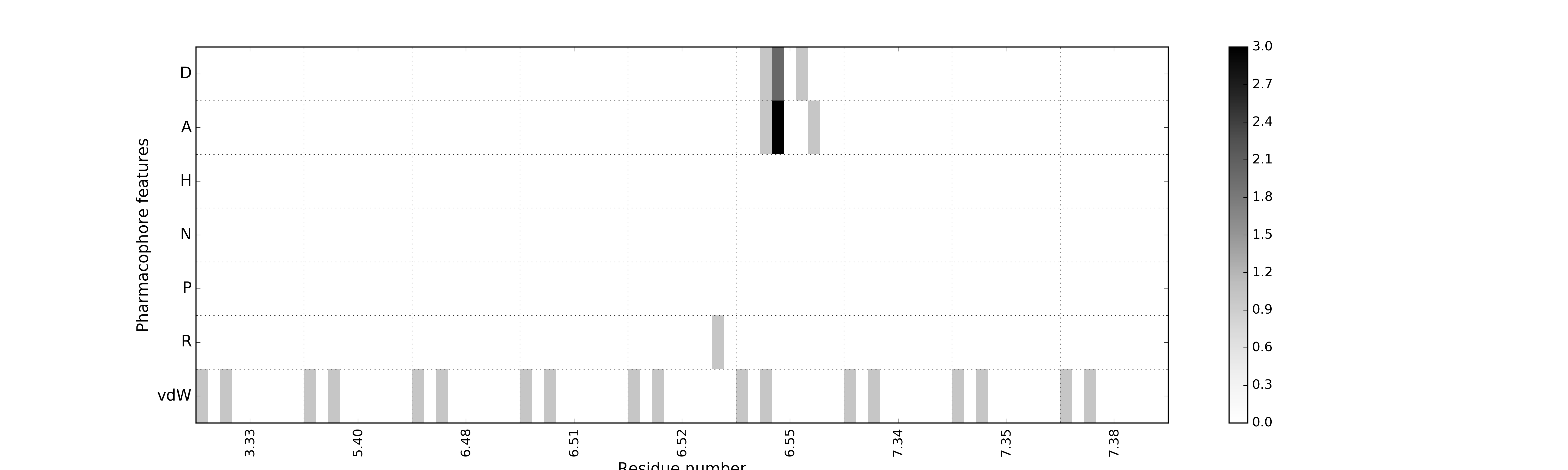 |
| 5-HT_1B_ receptor 4IAR | 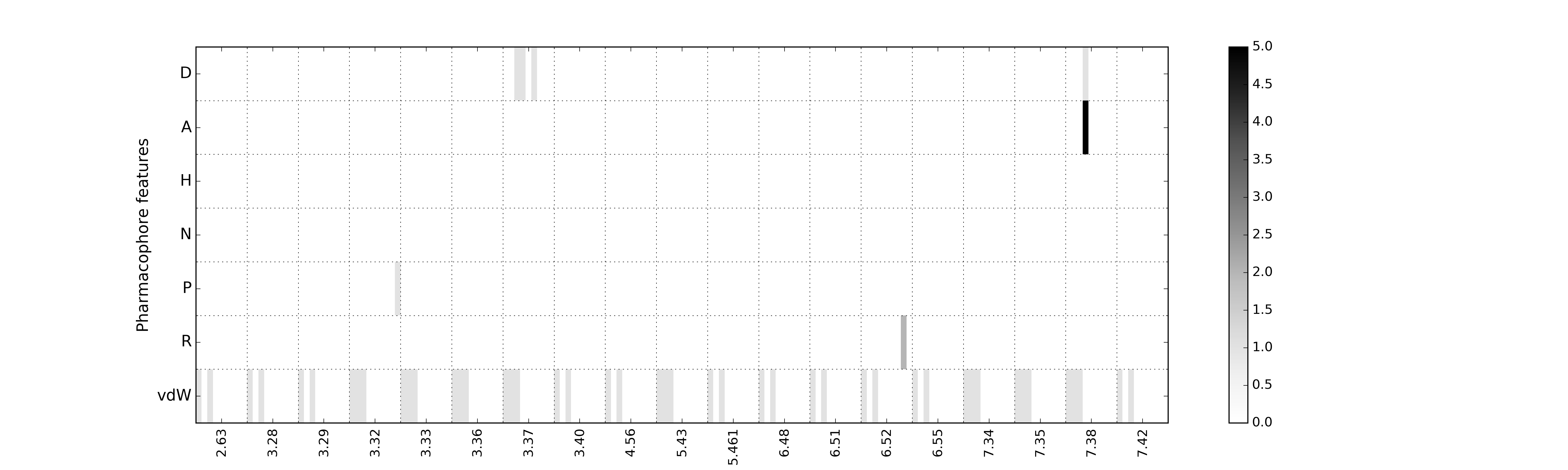 |
| 5-HT_2B_ receptor 4IB4 | 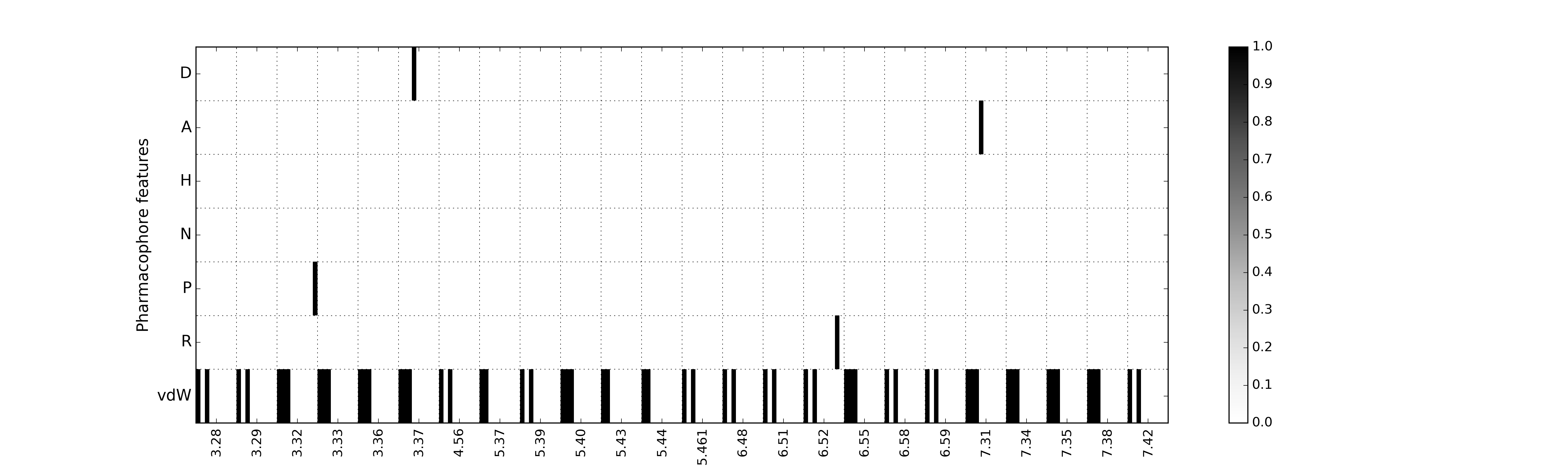 |
| CCR5 4MBS | 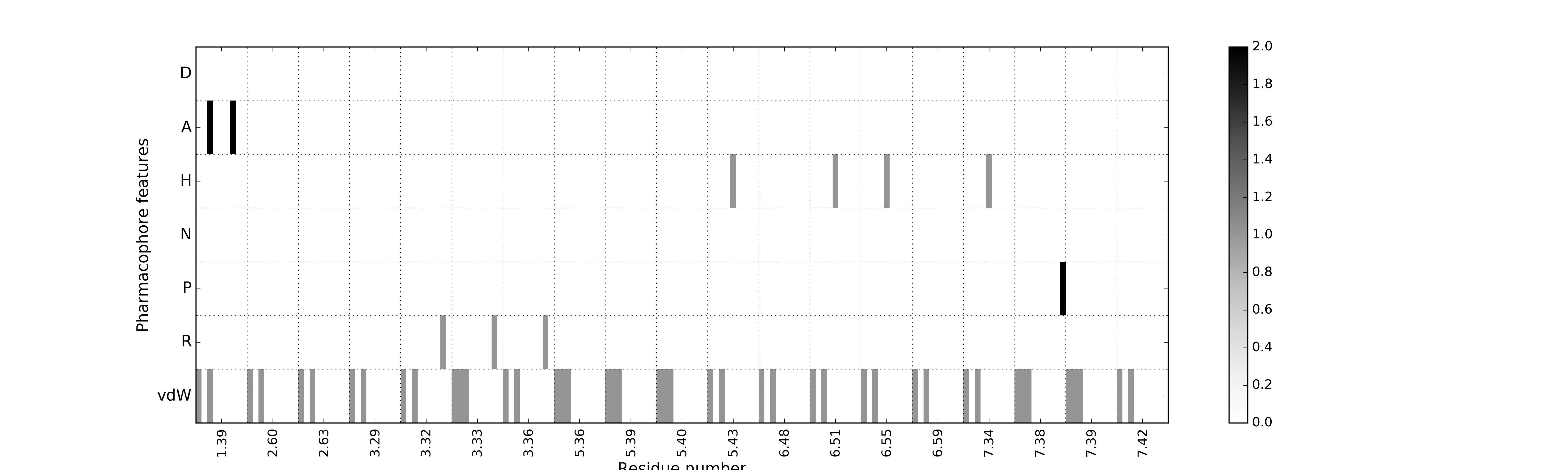 |
| δ receptor 4N6H | 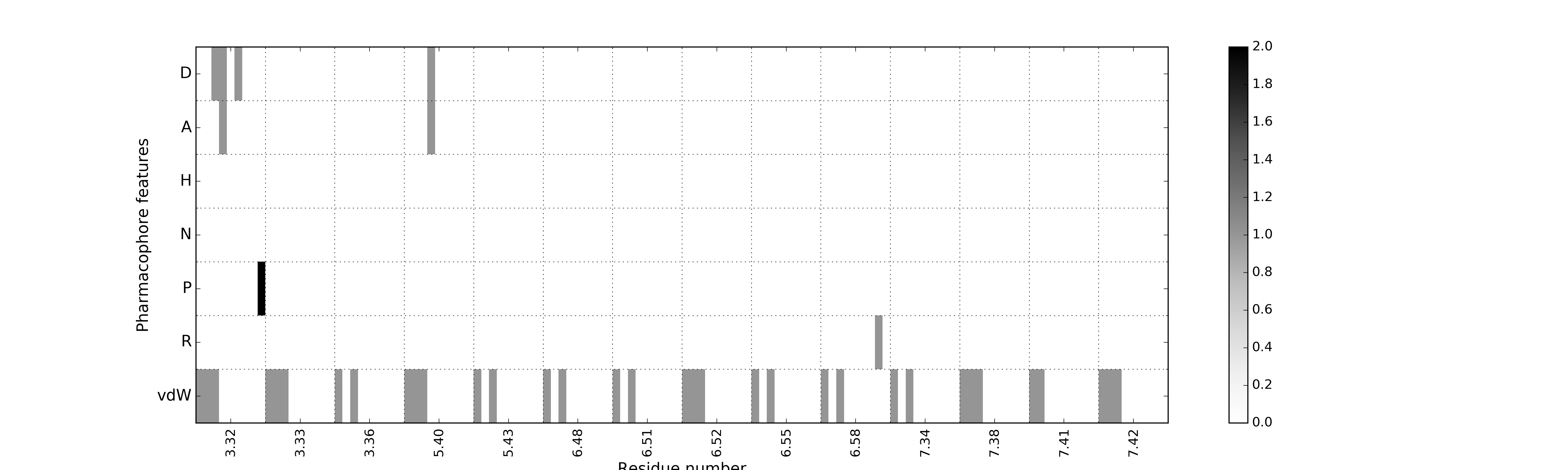 |
| FFA1 receptor 4PHU | 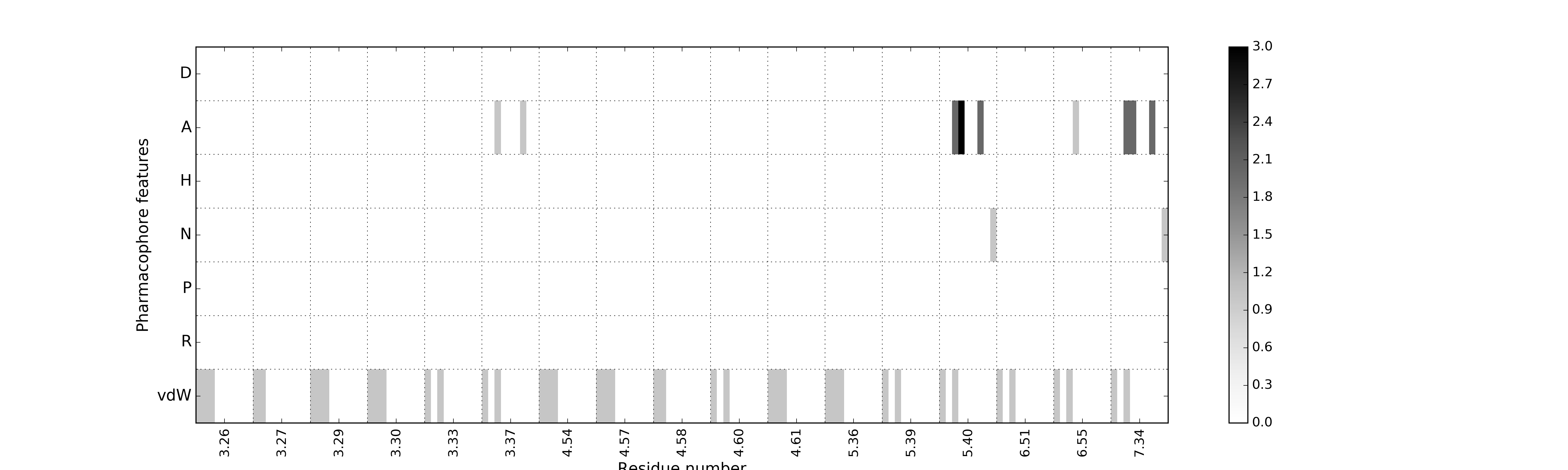 |
| P2Y_12_ receptor 4PZX | 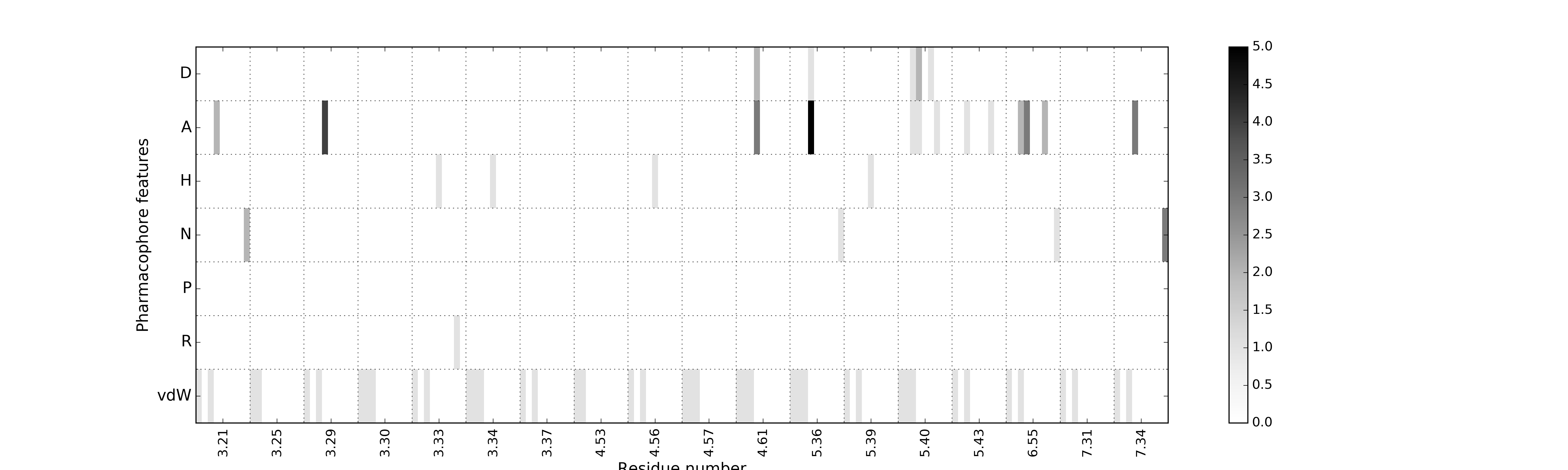 |
| OX_2_ receptor 4S0V | 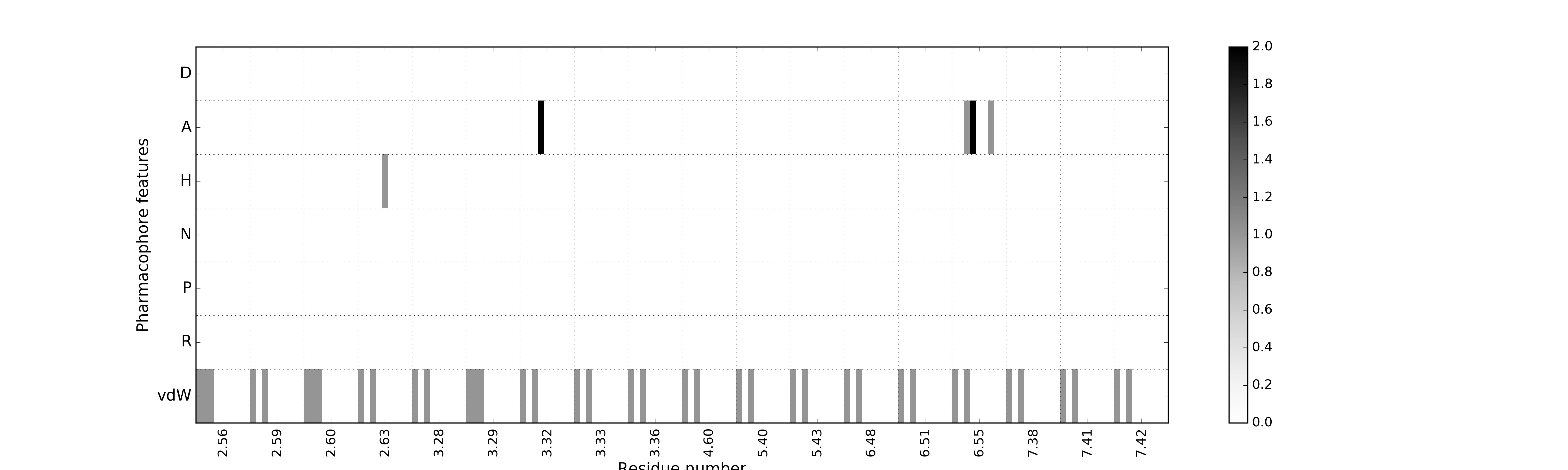 |
| M_3_ receptor 4U15 | 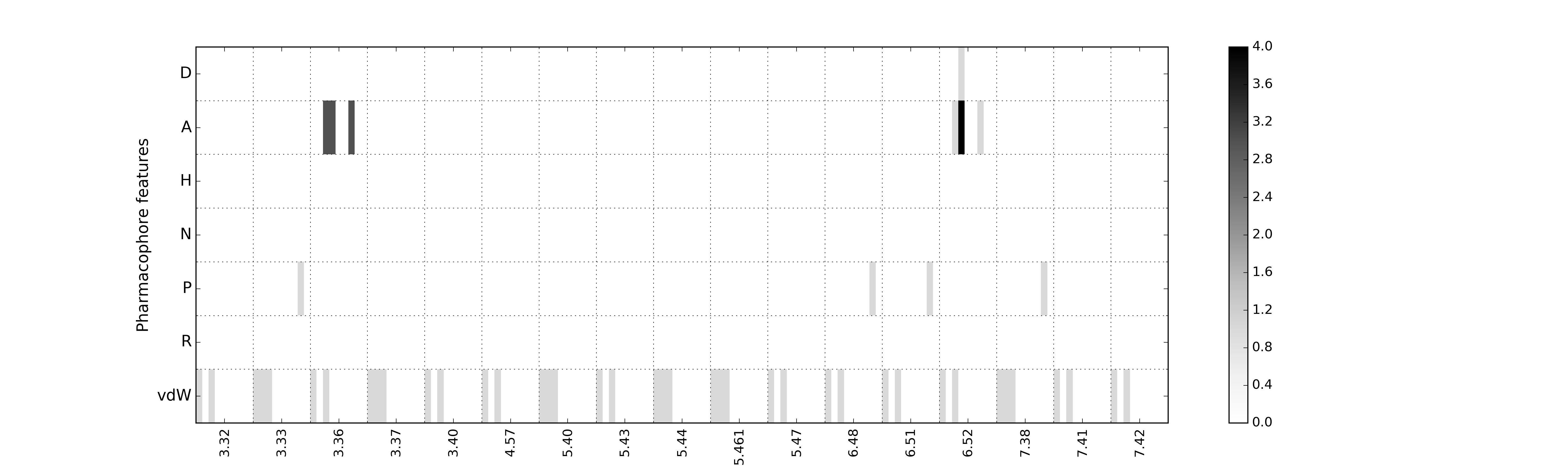 |
| P2Y_1_ receptor 4XNV | 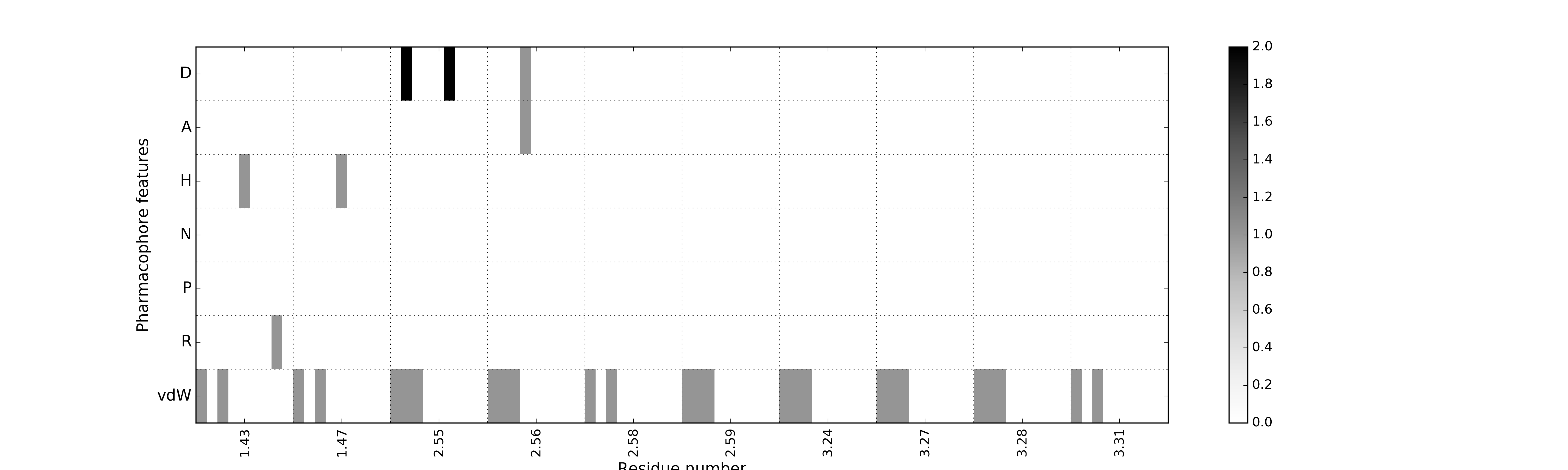 |
| AT_1_ receptor 4YAY | 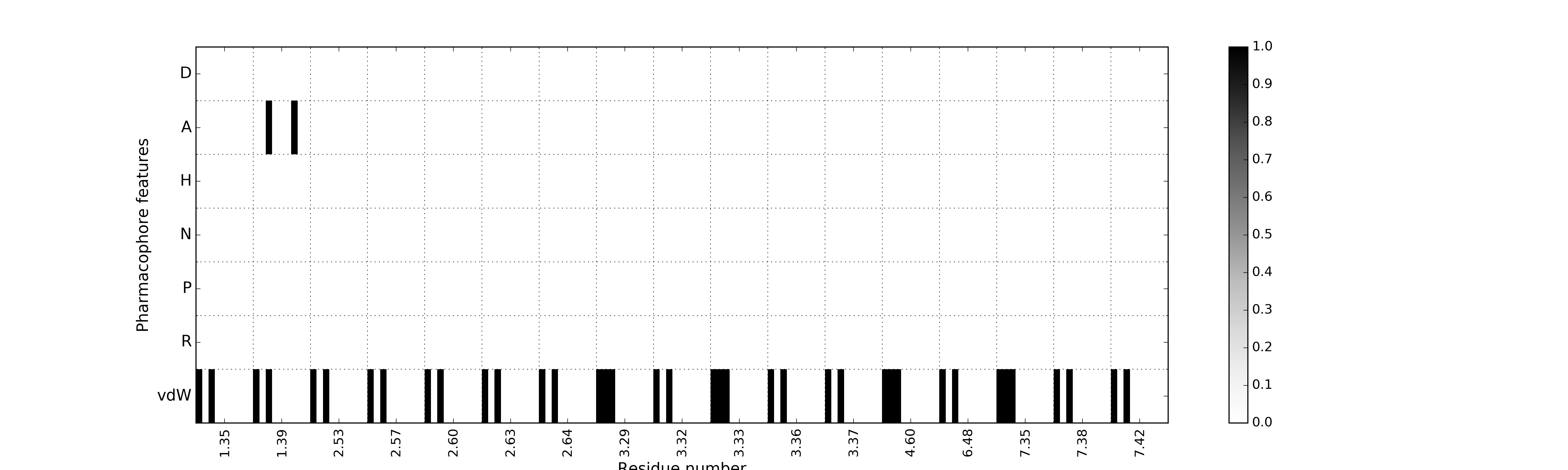 |
| LPA_1_ receptor 4Z35 | 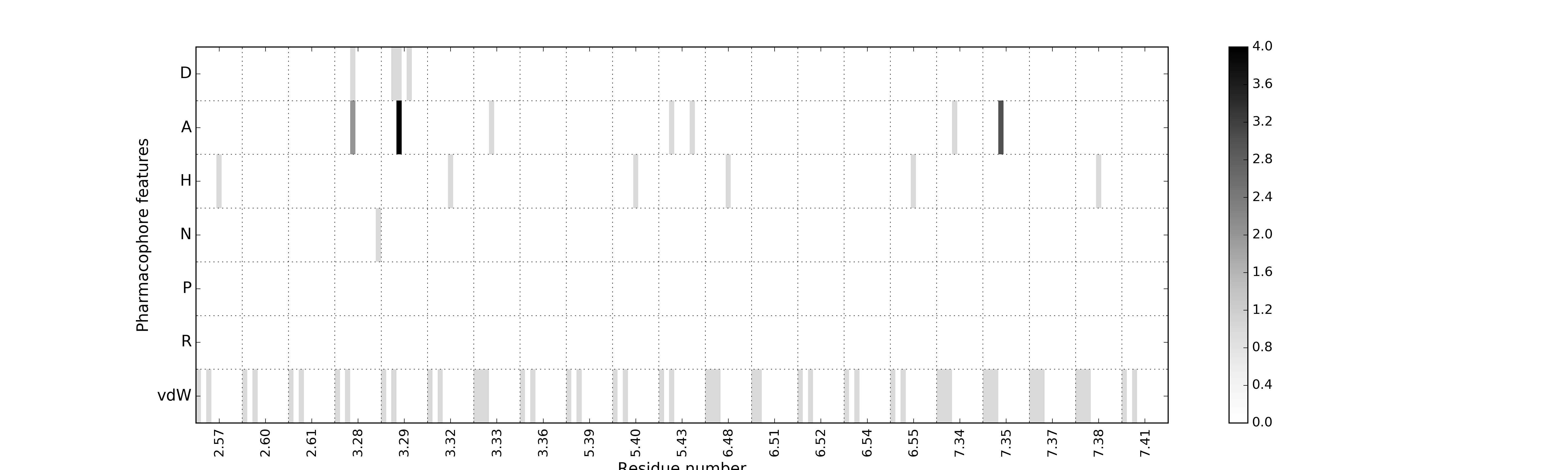 |
