# Supplementary figures and images for: 2D SIFt: a matrix of ligand-receptor interactions
Source: J Cheminform. 2021 Sep 8;13:66. doi: 10.1186/s13321-021-00545-9 (PMC8424890; doi:10.1186/s13321-021-00545-9)

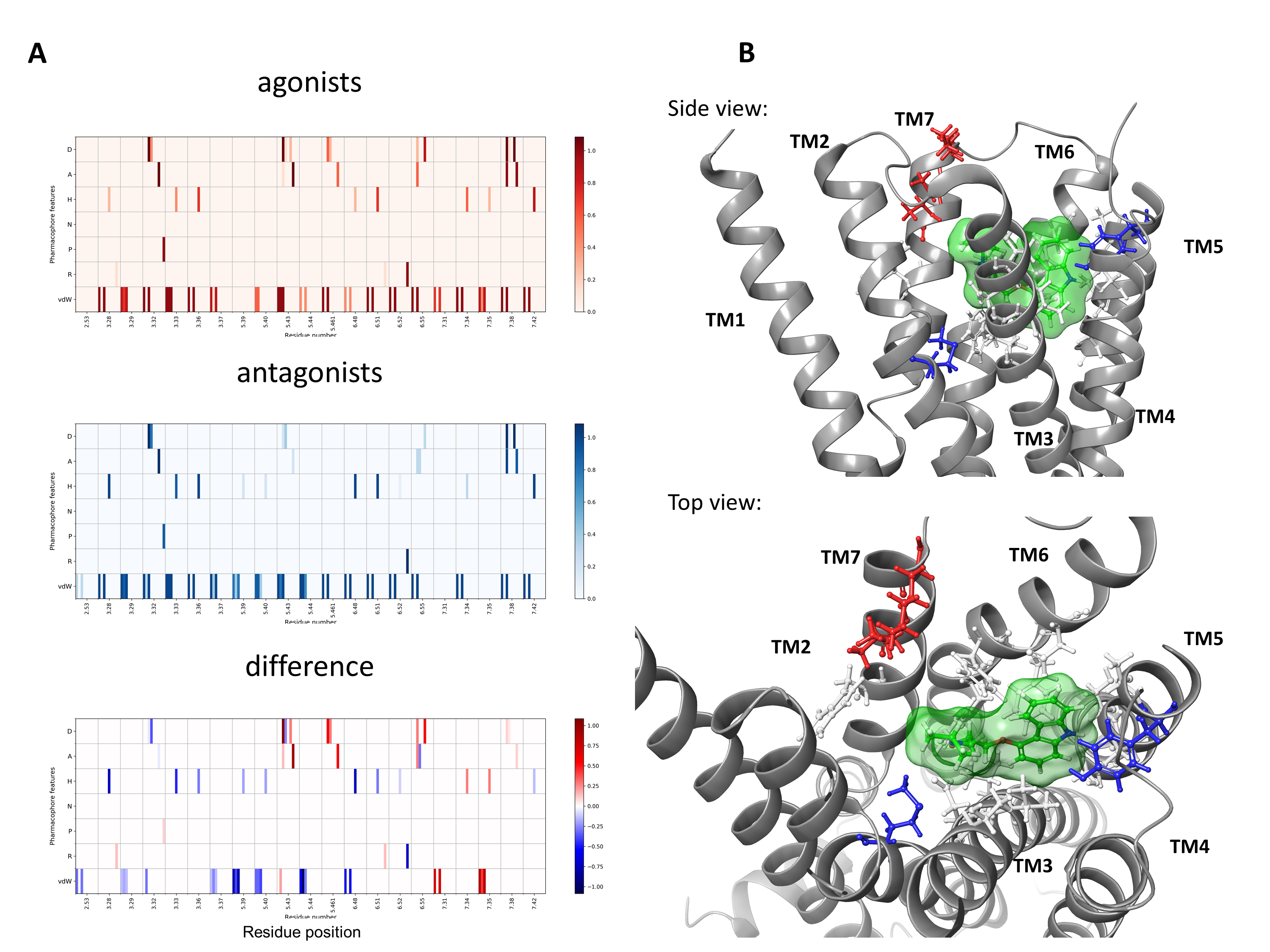

Supplement: Supplementary file 2 — Additional file 2: Figure S2. [file 13321_2021_545_MOESM2_ESM.tif]
